# *ARAF* Amplification in Small-Cell Lung Cancer-Transformed Tumors Following Resistance to Epidermal Growth Factor Receptor–Tyrosine Kinase Inhibitors

**DOI:** 10.3390/cancers16203501

**Published:** 2024-10-16

**Authors:** Ryo Kimura, Yuta Adachi, Kentaro Hirade, Satoru Kisoda, Shogo Yanase, Noriko Shibata, Makoto Ishii, Yutaka Fujiwara, Rui Yamaguchi, Yasuko Fujita, Waki Hosoda, Hiromichi Ebi

**Affiliations:** 1 Division of Molecular Therapeutics, Aichi Cancer Center Research Institute, Nagoya 464-8681, Japan; 2 Department of Respiratory Medicine, Nagoya University Graduate School of Medicine, Nagoya 466-8550, Japan; 3 Division of Cancer Systems Biology, Aichi Cancer Center Research Institute, Nagoya 464-8681, Japanr.yamaguchi@aichi-cc.jp (R.Y.); 4 Departments of Pathology and Molecular Diagnostics, Aichi Cancer Center Hospital, Nagoya 464-8681, Japan; nshibata@aichi-cc.jp (N.S.); whosoda@aichi-cc.jp (W.H.); 5 Department of Thoracic Oncology, Aichi Cancer Center Hospital, Nagoya 464-8681, Japan; 6 Division of Cancer Informatics, Nagoya University Graduate School of Medicine, Nagoya 466-8550, Japan; 7 Division of Advanced Cancer Therapeutics, Nagoya University Graduate School of Medicine, Nagoya 466-8550, Japan

**Keywords:** epidermal growth factor receptor tyrosine kinase inhibitor, resistance, small-cell lung cancer, transformation, *ARAF* amplification

## Abstract

Background/Objectives: Although tyrosine kinase inhibitors (TKIs) targeting EGFR-activating mutations significantly improved the outcome of EGFR-mutant NSCLC, resistance inevitably emerges. Despite the heterogeneity of these resistance mechanisms, many induce activation of MAPK signaling in the presence of EGFR-TKIs. While *ARAF* gene amplification is identified as a resistance mechanism that activates MAPK signaling by directly interacting with RAS, little is known about its clinicopathologic characteristics. Methods: We conducted a single-center retrospective analysis of the presence of *ARAF* amplification in re-biopsied samples in patients with EGFR-mutant NSCLC resistant to EGFR-TKIs. Demographic data, treatment course, and clinical molecular testing reports were extracted from electronic medical records. *ARAF* amplification was determined using a gene copy number assay. RNA sequence analysis was performed in patients with *ARAF* amplification as well as presenting histologic transformations to small-cell lung carcinoma (SCLC). Results: *ARAF* amplification was identified in five of ninety-seven patients resistant to erlotinib or gefitinib, and four of forty-eight patients resistant to Osimertinib. *ARAF* amplification was dominantly observed in female patients with EGFR exon 19 deletion. All *ARAF*-amplified tumors retained their founder EGFR mutation and were absent of secondary mutations. Two cases were found where *ARAF* amplification correlated with a histological transformation to SCLC. Conclusions: *ARAF* amplification was identified in 5–8% of EGFR-TKI-resistant tumors. The possible roles of ARAF in SCLC transformation warrant further investigation.

## Simple Summary

Despite the heterogeneity of resistance mechanisms to tyrosine kinase inhibitor (TKI)-targeting Epidermal Growth Factor Receptor (EGFR)-activating mutations, many induce the activation of MAPK signaling in the presence of EGFR-TKIs. *ARAF* gene amplification is identified as one such mechanism that activates MAPK signaling by directly interacting with RAS, yet its clinicopathologic characteristics remain poorly understood. In this study, we characterized nine cases with *ARAF* amplification resistant to EGFR-TKIs. Overall, these *ARAF*-amplified resistant tumors retained their original founder EGFR mutation and lacked secondary alterations. Furthermore, *ARAF* amplification was predominantly observed in female patients with EGFR exon 19 deletion. We also identified two cases showing a histologic transformation from lung adenocarcinoma to small-cell lung cancer (SCLC). *ARAF* amplification potentially fosters conditions that promote tumor cell survival and neuroendocrine marker transcription under EGFR-TKIs.

## 1. Introduction

The advent of targeted therapy with tyrosine kinase inhibitors (TKIs) directed against the epidermal growth factor receptor (EGFR) has led to dramatic improvements of response and progression-free survival (PFS) in patients with EGFR-mutated lung cancer. Nevertheless, essentially all initially responding patients eventually develop acquired resistance to the drugs. Mechanisms of resistance to EGFR-TKIs are classified into three major categories: (1) secondary mutations arising in EGFR (T790M, C797S), (2) activation of alternative pathways by another receptor (MET, HGF, AXL, IGF-1R) or downstream proteins (AKT mutations, loss of PTEN), and (3) phenotypic transformation [1,2].

While the first two resistance mechanisms are linked to gene alterations, the cause of phenotypic transformation remains elusive. Small-cell lung cancer (SCLC) transformation has been considered to be the most common phenotypic change in EGFR-mutant non-small-cell lung cancers (NSCLCs) during disease progression under treatment with earlier-generation EGFR TKIs. However, recent findings suggest that squamous cell carcinoma (SCC) transformation may be more prevalent (15%) among patients treated with osimertinib, based on a small cohort with limited follow-up [3]. Transformed tumors typically retain the original EGFR mutations [4].

A critical factor in diagnosing histologic transformation is the possibility of pre-existing mixed histology, which can be difficult to assess accurately with small biopsies or cytologic samples. This complexity underscores the challenges of studying histologic transformations. Additionally, while SCLCs often harbor mutations in or loss of the retinoblastoma gene (RB1) [5], RB1 loss is necessary but not sufficient for SCLC development from adenocarcinomas. Other pathways, such as NOTCH, Achaete-scute homolog 1 (ASCL1), and TP53, may also play significant roles [6]. The interplay between genetic and non-genetic resistance mechanisms during phenotypic transformation remains unclear.

In the presence of secondary mutations in EGFR or activation of bypass networks, EGFR-TKIs fail to suppress canonical cancer-signaling pathways, resulting in tumor cell proliferation. MAPK is a critical cancer-related pathway that plays pivotal roles in resistance to molecular targeted drugs [7,8]. In the MAPK pathway, RAF kinases are highly conserved serine/threonine kinases consisting of the three RAF isoforms: ARAF, BRAF, and CRAF. While BRAF alterations are predominant mechanisms of resistance to EGFR therapy [9,10], we recently identified that *ARAF* amplification causes resistance to EGFR inhibitors in EGFR mutant lung cancer [11]. Mechanistically, ARAF directly binds to RAS, displacing the GTPase-activating protein NF1, thereby antagonizing its inhibition of RAS. Therefore, increasing ARAF expression promotes MAPK signaling.

In this study, we analyzed the detailed characteristics of *ARAF*-amplified cancers that are resistant to first- or second-generation EGFR-TKIs, the prevalence of which we previously reported. Additionally, *ARAF* amplification was determined in patients with resistance to Osimertinib. We found that EGFR-TKI-resistant tumors with *ARAF* amplification were associated with female patients with EGFR exon 19 deletion. Furthermore, we identified *ARAF* amplification in small-cell lung cancer (SCLC)-transformed tumors and characterized their phenotype.

## 2. Materials and Methods

### 2.1. Patient Population

Ninety-seven EGFR-mutant NSCLC patients with acquired resistance to gefitinib or erlotinib from 2012 to 2016 and forty-eight EGFR-mutant NSCLC patients with acquired resistance to Osimertinib from 2016 to 2019 undergoing standard post-resistance biopsy of their tumor were identified at Aichi Cancer Center. Information on patient demographics, previous lines of therapy, and survival were collected until January 2024. The study was approved by the Institutional Review Board at Aichi Cancer Center (approved #R011093), and all research was performed in accordance with relevant guidelines/regulations. Informed consent was obtained from all subjects.

### 2.2. Gene Copy Number Analysis

DNA was extracted from formalin-embedded paraffin samples using the DNeasy Blood and Tissue Kits (QIAGEN, Hilden, Germany) from the re-biopsied samples. The DNA from re-biopsied samples were screened for *ARAF* amplification using a TaqMan gene copy number assay (assay ID: Hs05691600_cn, Applied Biosystems, Waltham, MA, USA). TaqMan Copy Number Reference Assay human RNase P was used as the endogenous reference gene. The fold increase in copy number was calculated as the ratio of the *ARAF* signal in each tumor to that obtained in the normal *ARAF* gene in human genomic DNA (Promega, Madison, WI, USA). Tumors harboring an *ARAF* copy number of 5.0 or higher were defined as positive for gene amplification.

### 2.3. Immunohistochemistry

Tumor tissues were fixed in 4% paraformaldehyde overnight and stored in 70% ethanol before being embedded in paraffin and sectioned at a thickness of 4 μm. Sections were deparaffinized in Hemo-De (FALMA, Tokyo, Japan) and rehydrated in successive ethanol baths. Sections were incubated with primary antibodies overnight at 4 °C: TTF-1 (M3575, DAKO, Agilent Technologies, Santa Clara, CA, USA), CD56 (MA5-16446, ThermoFisher, Waltham, MA, USA), synaptophysin (MA5-14532, ThermoFisher), YAP1 (sc-101199, Santa Cruz, Dallas, TX, USA), Neuro D1 (ab205300, Abcam, Cambridge, UK), and p40 (07394420001, Roche, Basel, Switzerland). After incubation with peroxidase conjugated secondary antibodies, detection was performed with the DAB technique (Vector Laboratories, Newark, CA, USA), followed by nuclear counterstaining with hematoxylin.

### 2.4. RNA Sequencing and Gene Set Enrichment Analysis

Total RNA was extracted from FFPE samples using the RNeasy Mini Kit. An RNA-seq library was prepared using the SMARTer^®^ Stranded Total RNA-Seq Kit v3 -Pico Input Mammalian (Takara Bio, Shiga, Japan) following the manufacturer’s protocol. The enriched libraries were sequenced as 150 bp paired-end reads using NovaSeq 6000 (Illumina. Inc., San Diego, CA, USA). Normalized gene-level expression values were estimated as follows: First, fastp (v0.23.4) [12] software for trimming adapter sequences from sequencing reads was applied to paired-end RNA-seq fastq files for each sample. Second, ribosomal RNA-derived reads were removed using RiboDetector (https://github.com/hzi-bifo/RiboDetector, accessed on 7 February 2024). Third, kallisto (v0.46.2) [13] software was used to estimate a transcript-level abundance from the trimmed reads with known transcript sequences and annotations in GENCODE Release 33 [14]. Third, gene-level abundance values in transcripts per million (TPM) were estimated by summarizing the transcript-level estimates with the R package tximport (v1.22.0) in the R environment (v4.1.2) using a matching list between transcript IDs and gene IDs based on the annotations of GENCODE. TPM for each gene is shown in Supplementary Table S1. Gene set enrichment analysis (GSEA) was performed for the gene-list ordered by the log2FC values using the R package fgsea (v1.20.0) with gene-set collections in MSigDB (v7.5.1) [15], where one was added to each TPM value to calculate the corresponding log2FC value.

## 3. Results

### 3.1. ARAF Amplification in Patients with Resistance to First-Generation EGFR-TKIs

**Figure 1:**
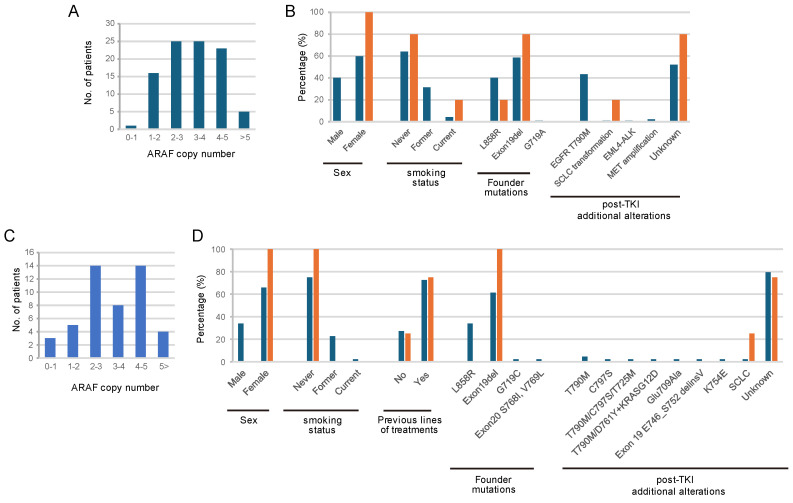
The distribution of *ARAF* copy numbers and clinicopathologic characteristics is shown for patients resistant to first– and second–generation EGFR-TKIs (**A**,**B**) and Osimertinib (**C**,**D**). Figure 1B and Figure 1D provide the percentages of each characteristic within their respective categories. Blue represents *ARAF* non-amplified cases, while orange indicates *ARAF* amplified cases.

*ARAF* amplification was identified in five of ninety-seven patients whose tumors were re-biopsied after developing resistance to erlotinib or gefitinib. The distribution of *ARAF* copy number is shown in Figure 1A. The median age was similar in patients with and without *ARAF* amplification, at 65 years (range: 36–85) for *ARAF*-amplification-negative cases and 66 years (range: 36–80) for *ARAF*-amplification-positive cases. Clinicopathological characteristics of *ARAF*-amplified patients is summarized in Figure 1B. *ARAF* amplification was dominantly observed in female patients with deletion of EGFR exon 19. All *ARAF*-amplified tumors retained their original EGFR mutation and were absent from T790M. Tumors before TKI treatment were available for three cases and showed no evidence of *ARAF* amplification.

### 3.2. ARAF Amplification in Patients with Resistance to Third-Generation EGFR-TKIs

To interrogate whether *ARAF* amplification is associated with resistance to third-generation EGFR-TKIs, we screened 48 re-biopsied specimens following resistance to Osimertinib. Among them, 13 patients were treated with Osimertinib as first-line treatment. The distribution of *ARAF* copy number is shown in Figure 1C, and *ARAF* amplification was identified in four cases. The median age was similar in patients with and without *ARAF* amplification, at 63 years (range: 37–83) for *ARAF* amplification-negative cases, and 70 years (range: 51–82) for *ARAF* amplification-positive cases. All *ARAF* amplified tumors retained their founder EGFR mutation and were absent of C797S (Figure 1D). The absence of *ARAF* amplification before Osimertinib treatment was confirmed in three cases.

### 3.3. SCLC Transformation in ARAF-Amplified Resistant Tumors

We have identified two SCLC-transformed cases among *ARAF*-amplified tumors. In patients resistant to 1st/2nd generation EGFR-TKIs, SCLC transformation occurred in 1 out of 92 patients without *ARAF* amplification and 1 out of 5 patients with *ARAF* amplification (*p* = 0.10). In those resistant to Osimertinib, SCLC transformation was seen in 1 out of 44 patients without *ARAF* amplification and 1 out of 4 patients with *ARAF* amplification (*p* = 0.16). When combining these results, SCLC transformation was observed in 2 out of 136 patients without *ARAF* amplification and 2 out of 9 patients with *ARAF* amplification (*p* = 0.018).

**Figure 2:**
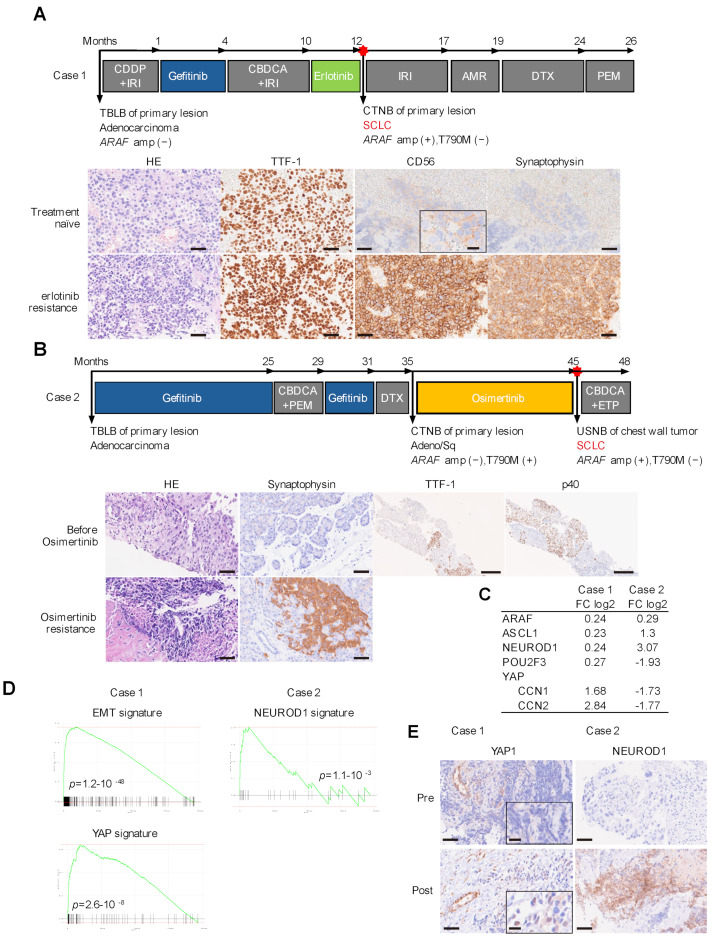
Cases with *ARAF* amplification in SCLC-transformed tumors following resistance to EGFR-TKI. (**A**) Case with SCLC transformation after resistance to erlotinib treatment. A pre-treatment sample was taken at diagnosis using transbronchial lung biopsy (TBLB), and re-biopsied samples were taken at resistance to erlotinib treatment using CT-Guided needle biopsy (CTNB). Scale bar, 100 µm for low-power field and 50 µm for high-power field in CD56. IRI, irinotecan; AMR, amrubicin; DTX, docetaxel; PEM, pemetrexed. (**B**) Case with SCLC transformation after resistance to Osimertinib treatment. A pre-treatment sample was taken after resistance to gefitinib using CTNB, and re-biopsied samples were taken at resistance to Osimertinib using ultrasound-guided needle biopsy (USNB). Scale bar, 100 µm. ETP, etoposide. (**C**) Log 2-fold change in expression of the indicated genes at the time of resistance compared to the pre-treatment biopsy samples shown in (**A**,**B**). (**D**) GSEA data for the indicated signatures. (**E**) Immunohistochemistry staining for indicated antibodies. Scale bar, 100 µm for low-power field and 50 µm for high-power field in YAP.

In the first-generation EGFR-TKI resistance cohort, a 74-year-old female was diagnosed with TTF-1-positive adenocarcinoma. Some cells were positive for CD56, but negative for synaptophysin (Case 1, Figure 2A). Blood tests showed an increase in CEA and neuron-specific enolase (NSE), which were 25.7 ng/mL and 46.2 ng/mL, respectively. The patient was treated with platinum-based chemotherapy followed by EGFR-TKIs due to the elevation of neuroendocrine tumor makers. Neither gefitinib nor erlotinib were effective. Re-biopsy of the primary lesion identified that most of the tumor cells were CD56 and synaptophysin positive. Genetic tests showed both exon 19 deletion and *ARAF* amplification.

In the Osimertinib-resistant cohort, an 82-year-old female was originally diagnosed with an adenocarcinoma harboring EGFR exon 19 deletion. She achieved a partial response with gefitinib, followed by cytotoxic agents at progression. The biopsy before Osimertinib treatment showed a heterogenous pattern of TTF-1 expression. TTF-1-negative cells were positive for p40, suggesting adenosquamous carcinoma (Case 2, Figure 2B). Genetic tests identified founder exon 19 deletion, but not T790M or *ARAF* amplification. Biopsied samples after resistance to Osimertinib showed SCLC transformation with an elevation of the *ARAF* copy number from 3.5 to 7.9.

Furthermore, a 35-year-old female with an undifferentiated adenocarcinoma provided a possible connection between *ARAF* amplification and SCLC transformation. A re-biopsied sample after resistance to gefitinib identified CD56-positive cells, while adenocarcinoma histology was also observed, suggesting combined SCLC with adenocarcinoma. The sample was negative for *ARAF* amplification; however, the biopsied tumor after disease progression with etoposide and cisplatin followed by erlotinib identified *ARAF* amplification (Supplementary Figure S1).

While there are no available large datasets to determine the prevalence of *ARAF* amplification in SCLC-transformed tumors following EGFR-TKI treatment, we interrogated the prevalence of *ARAF* amplification in a large clinical sequencing cohort including 293 SCLC samples, identifying *ARAF* amplification in 1 of 9 cases of SCLC with EGFR-activating mutations, compared to only 1 of 284 cases of EGFR wildtype SCLC (*p* = 0.06 by Fisher’s test) [16].

### 3.4. SCLC Subtypes in Resistant Tumors

SCLC can be classified into four subtypes based on transcriptional signatures driven by specific transcription factors: achaete-scute homolog 1 (ASCL1), neurogenic differentiation factor 1 (NEUROD1), yes-associated protein 1 (YAP1), and POU class 2 homeobox 3 (POU2F3). These subtypes are designated as SCLC-A (ASCL1-dominant), SCLC-N (NEUROD1-dominant), SCLC-P (POU2F3-dominant), and SCLC-Y (YAP1-dominant) [17,18]. To estimate the subtypes of these resistant tumors, RNA sequencing analysis was performed in paired samples before and after treatment with EGFR-TKIs. Among representative genes for each subtype, YAP downstream CCN1 (CTGF) and CCN2 (CYR61) were upregulated in case 1 (Figure 2C). GSEA showed enrichment of epithelial–mesenchymal transition (EMT)-related genes and YAP pathway genes, suggesting SCLC-Y subtype (Figure 2D). The Osimertinib-resistant tumor showed upregulation of NEUROD1 expression (Figure 2C). Furthermore, the NEUROD1-related gene signature was the most significantly enriched among 830 cell type signature gene sets, suggesting a SCLC-N subtype (Figure 2D). Consistently, post-treatment samples became positive for YAP1 and NeuroD1, respectively (Figure 2E).

## 4. Discussion

In our cohort, *ARAF* amplification was identified in 5–8% of EGFR-TKI-resistant tumors. All tumors retained their original EGFR mutation and were absent of secondary alterations such as T790M and C797S. *ARAF* amplification was principally observed in female patients with EGFR exon 19 deleted tumors, while the emergence of T790M and C797S were not associated with clinical characteristics, including age, sex, or primary mutation status [19,20]. The female predominance may be associated with the localization of *ARAF* in Xp11.3. Among *ARAF*-amplified cancers, we identified two cases with histologic transformation from adenocarcinoma to SCLC.

Lineage plasticity—a cells’ capacity to switch from one predetermined developmental trajectory to another—is suggested to contribute to intratumor heterogeneity and serve as a source of adaptation to the pressures of targeted therapy [21]. Histologic transformation from adenocarcinoma to SCLC arises in 4–15% of patients with EGFR-mutant NSCLC that develops an acquired resistance to first- and third-generation EGFR-TKIs [4,22]. Consistent with *ARAF*-amplified SCLC-transformed cases, SCLC transformation predominantly occurs in tumors with EGFR exon 19 deletion, with transformed tumors maintaining their founder mutations [4,22].

The precise mechanism of SCLC transformation is not fully elucidated. While neuroendocrine tumors arising from distinct epithelial tissues seem to require modulation of transcriptional and epigenetic programs [23,24], MAPK signaling diminished the expression of transcription factors needed for neuroendocrine cell traits [25]. Therefore, inhibition of MAPK signaling by EGFR-TKI may facilitate lineage switching in EGFR-mutant lung cancer cells. While activated MAPK signaling suppresses neuroendocrine lineage differentiation, concomitant overexpression of MYC or BCL2 accelerates neuroendocrine tumor formation [24]. While MYC is a well-known downstream target of MAPK, the MAPK pathway also regulates BCL2 family proteins by inactivating pro-apoptotic BIM and stabilizing anti-apoptotic MCL1 [26]. We previously demonstrated that drug-tolerant persister cells in EGFR-TKI treatment increased mTORC1-mediated mRNA translation of MCL-1 [27]. These suggest that sustained MAPK activation is also necessary to maintain cell viability. Notably, ARAF has a significantly lower kinase activity than BRAF and CRAF, but its overexpression prolonged the duration of MAPK activation [11], potentially creating conditions conducive to tumor cell survival and the transcription of neuroendocrine markers in the presence of EGFR-TKIs.

Our study is limited by the small number of patients due to the rarity of SCLC transformation following EGFR-TKI treatment. Another drawback is that we could not determine where ARAF protein expressed due to a lack of validated antibodies. Lastly, the retrospective nature of the present series needs confirmation by larger cohort studies. Despite these limitations, it is warranted to pursue the role of *ARAF* amplification in the pathogenesis of SCLC transformation in EGFR-TKI-treated cancers.

## 5. Conclusions

In this study, we identified *ARAF* amplification in EGFR-TKI-resistant tumors. These *ARAF*-amplified resistant tumors retained their original founder EGFR mutation and lacked secondary alterations. Furthermore, *ARAF* amplification was predominantly observed in female patients with EGFR exon 19 deletion. Notably, we also identified two cases showing histologic transformation from lung adenocarcinoma to SCLC. *ARAF* amplification potentially fosters conditions that promote tumor cell survival and neuroendocrine marker transcription under EGFR-TKIs, particularly in female patients with EGFR exon 19 deletion.

## Data Availability

The datasets generated and/or analyzed during the current study are available in the Gene Expression Omnibus repository, GSE274293.

## Supplementary Materials

The following supporting information can be downloaded at: https://www.mdpi.com/article/10.3390/cancers16203501/s1, Figure S1: A case of *ARAF* amplification acquired following erlotinib treatment; Table S1. RNA sequence results of paired samples.
