# Supplementary material for: ARAF Amplification in Small-Cell Lung Cancer-Transformed Tumors Following Resistance to Epidermal Growth Factor Receptor–Tyrosine Kinase Inhibitors
Source: Cancers (Basel). 2024 Oct 16;16(20):3501. doi: 10.3390/cancers16203501 (PMC11506424; doi:10.3390/cancers16203501)
Supplement: Supplementary file 1 [file cancers-16-03501-s001.zip › Supplementary Figure S1.pdf]

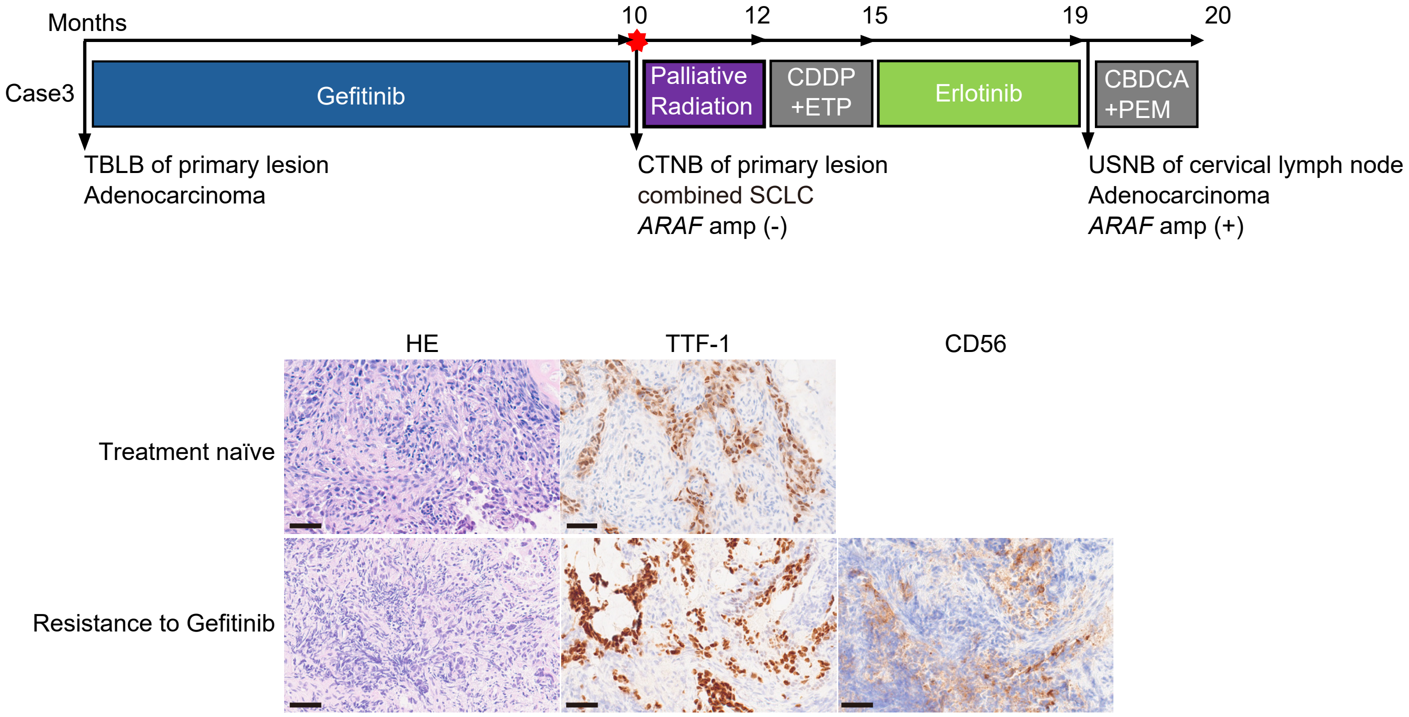


**Supplementary Figure S1. A case of *ARAF* amplification acquired following erlotinib treatment.**

This patient was diagnosed with adenocarcinoma by pleural effusion. At resistance to gefitinib treatment, re-biopsied samples from CT-Guided needle biopsy (CTNB) identified mixed histology with TTF-1 positive cells and CD56 positive cells, suggesting combined small cell carcinoma. The patient was treated with platinum-based chemotherapy followed by erlotinib. At resistance to erlotinib treatment, ultrasound-guided needle biopsy (USNB) from the cervical lymph node showed *ARAF* amplification. Notably, the histology was TTF-1 positive adenocarcinoma without SCLC cells.
